# Voltammetric Sensor for Doxorubicin Determination Based on Self-Assembled DNA-Polyphenothiazine Composite

**DOI:** 10.3390/nano13162369

**Published:** 2023-08-18

**Authors:** Anastasiya Malanina, Yurii Kuzin, Alena Khadieva, Kseniya Shibaeva, Pavel Padnya, Ivan Stoikov, Gennady Evtugyn

**Affiliations:** 1 A.M. Butlerov Chemistry Institute, Kazan Federal University, 18 Kremlevskaya Street, Kazan 420008, Russia; 2 Analytical Chemistry Department, Chemical Technology Institute, Ural Federal University, 19 Mira Street, Ekaterinburg 620002, Russia

**Keywords:** DNA sensor, electropolymerization, self-assembling, phenothiazine electropolymerization, DNA intercalation

## Abstract

A novel voltammetric sensor based on a self-assembled composite formed by native DNA and electropolymerized N-phenyl-3-(phenylimino)-3H-phenothiazin-7-amine has been developed and applied for sensitive determination of doxorubicin, an anthracycline drug applied for cancer therapy. For this purpose, a monomeric phenothiazine derivative has been deposited on the glassy carbon electrode from the 0.4 M H_2_SO_4_-acetone mixture (1:1 *v*/*v*) by multiple potential cycling. The DNA aliquot was either on the electrode modified with electropolymerized film or added to the reaction medium prior to electropolymerization. The DNA entrapment and its influence on the redox behavior of the underlying layer were studied by scanning electron microscopy and electrochemical impedance spectroscopy. The DNA–doxorubicin interactions affected the charge distribution in the surface layer and, hence, altered the redox equilibrium of the polyphenothiazine coating. The voltametric signal was successfully applied for the determination of doxorubicin in the concentration range from 10 pM to 0.2 mM (limit of detection 5 pM). The DNA sensor was tested on spiked artificial plasma samples and two commercial medications (recovery of 90–95%). After further testing on real clinical samples, the electrochemical DNA sensor developed can find application in monitoring drug release and screening new antitumor drugs able to intercalate DNA.

## 1. Introduction

Doxorubicin is an anthracycline drug commonly applied in solid tumor therapy. It was approved for medical application and involved in the WHO List of Essential Medicines [1]. Doxorubicin’s effect involves its intercalation in the double-stranded DNA helix followed by suppression of biochemical functions, e.g., transcription and replication required for the DNA biosynthesis [2]. It can also cause the generation of reactive oxygen species in mitochondria by the reaction with dissolved oxygen [3]. The efficiency of doxorubicin is limited by frequently observed side effects, of which delayed cardiomyopathy, acute life-threatening inflammation of the bowel, and dermatological disorders are most commonly mentioned [4,5,6]. This calls for the effective monitoring of the doxorubicin levels in biological fluids and establishing personal doses of medication depending on its release from the organism. Traditionally, optical [7,8] and chromatographic [9,10] methods of analysis are used for this purpose.

Many efforts have been made in the development and application of new nanomaterials for extending optic detection systems. Thus, specially designed nanophotonic structures have been synthesized and ascribed with complex mathematical functions applicable for sensing elements [11]. Core@shell nanoparticles with an Ag core covered with a silica layer showed absorption in the UV–Vis spectrum that depended on the thickness of the shell [12]. The size and optical properties of the metamaterials obtained can be directly varied by the choice of precursor using the microwave polyol method [13]. The effect can be used for sensitive detection of many species affecting the optic phenomena on the nanoparticles surface. Epsilon-near-zero (ENZ) metamaterials with a narrow metallic waveguide channel are able to detect changes in the permittivity and refractive index of the dielectric microenvironment as a universal approach to the characterization of homogeneous materials [14]. Furthermore, graphene materials offer both optic and electrochemical properties related to the influence of analytes on electromagnetic properties of the materials able to detect DNA, recombinant proteins, and antibodies [15].

Being universal and sensitive, optical methods based on the detection of electromagnetic properties near the sensor interface need sophisticated data treatment and complicated protocols for material synthesis. Electrochemical approaches are more adaptive to the point-of-care testing conditions, including drug residue detection [16,17,18].

Electrochemical detection techniques are considered as a fast, inexpensive, and reliable alternative to more sophisticated universal instrumentation, Nevertheless, to reach the sensitivity of analysis comparable with chromatography, conventional electrodes should be modified with effective mediators of electron transfer that both amplify the currents recorded and diminish the working potential. The latter condition is important for the direct determination of analytes in complex media containing oxidizable organic species, e.g., biological fluids. Searching for new mediators and characterization of their implementation in the assembly of electrochemical sensors is one of the modern trends of electroanalytical chemistry. In the case of biosensors, such mediators can also provide effective immobilization of the biorecognition elements in the surface layer.

Phenothiazine derivatives have received a wide application in biochemistry and analytical chemistry as specific dyes exert reversible redox properties and are able to concentrate in the cells and organelles. They also exert antipsychotic, antimalarial, insecticidal, antifungal, antibacterial, and anthelmintic properties [19,20,21]. The redox activity of phenothiazines used as drugs has been applied for their direct electrochemical determination [22,23,24]. Monomeric dyes and the products of their electropolymerization were also tested as mediators and showed high electroactivity and efficiency as mediators and electrocatalysts [25,26,27,28,29,30,31,32]. 

Although many phenothiazine derivatives are easily adsorbed on the bare electrodes, their electropolymerization has additional advantages in sensor assembling, e.g., easy control of the reaction by the potential applied, variation in the quantities of the accumulated products on the electrode interface, no auxiliary reagents required in chemical polymerization (chemical oxidants, etc.), and the possibility of combining the deposition of redox active components and of the biopolymer entrapped in the growing polymer film [33]. Analytical application of electropolymerized materials started from polyaniline [34] and polypyrrole [35]; recently the attention of researchers was turned to the electropolymerization of phenazines, phenothiazines, and phenoxazines that can be performed in the absence of organic solvents and in milder conditions [36,37,38,39,40]. As examples, electropolymerization of thionine [41], methylene blue [42], methylene green [43], azure A [44], and azure B [45] can be mentioned. Appropriate polymeric products improved the conditions of the electron exchange and demonstrated catalytic activity in the conversion of hydrogen peroxide [46] and NADH [47]. Meanwhile, spontaneous aggregation and limited solubility of the monomeric dyes affects the regularity of the polymer films obtained and the reproducibility of the modifier properties. Further improvement of the analytical and operational characteristics of electrochemical sensors calls for searching for new derivatives and their testing in the assembly of biosensors for drugs selection, in solar batteries, and in charge storage devices [48,49,50,51,52].

Recently, we have studied the electrochemical behavior of N-phenyl-3-(phenylimino)-3H-phenothiazin-7-amine (PhTz) with electropolymerization, alone and in the assembly of the DNA sensor [53]. Phenyl imine fragments present in its structure allow two mechanisms of polymerization, including the one proposed for polyaniline in strong acidic media [54,55,56] and another one attributed to the phenothiazine core. In this work, we have for the first time polymerized the PhTz from the acidic media and showed intrinsic redox activity of the product (polyPhTz) in the assembly of the DNA sensor and in the presence of doxorubicin as a model intercalator. 

## 2. Materials and Methods

**Figure 1:**
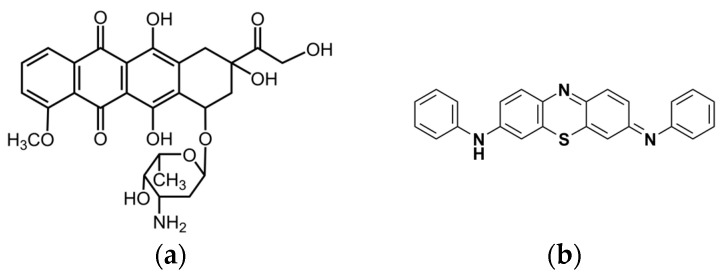
Chemical structures of doxorubicin (**a**) and N-phenyl-3-(phenylimino)-3H-phenothiazin-7-amine (**b**).

Low-molecular DNA from salmon testes (DNA1, <5% protein, A_260/280_ = 1.4), from fish sperm (DNA2, mol. weight 40–1000 kDa), and from chicken erythrocytes (DNA3, “Reanal”, Budapest, Hungary, average mol. mass 1.2 MDa), doxorubicin hydrochloride (98–102%) (Figure 1a), idarubicin hydrochloride (>98%), daunorubicin hydrochloride (>90%), valrubicin (<100%), cyclophosphamide (EP reference standard), prednisone (dehydrocortisone, >98%), dacarbazine (EP reference standard), potassium hexacyanoferrate (III) (99%), potassium hexacyanoferrate (II) trihydrate (98.5–102%), and HEPES (4-(2-hydroxyethyl)-1-piperazineethanesulfonic acid) were purchased from Sigma-Aldrich, Dortmund, Germany. All solutions were prepared with Millipore Q^®^ water (Simplicity^®^ water purification system, Merck-Millipore, Mosheim, France). Doxorubicin-TEVA^®^ and Doxorubicin-LANS^®^ (lyophilizates for intravascular injection solutions) were purchased at a local pharmacy market.

The PhTz (chemical structure in Figure 1b was synthesized as described elsewhere [57]. For electropolymerization, it was dissolved in acetone and then mixed with 0.4 M H_2_SO_4_ in 1:1 (*v*/*v*) ratio. In electropolymerization, the working concentration of PhTz (72 μM) corresponded to its maximal solubility. DNA was dissolved in deionized water. 

### 2.1. Apparatus

Electrochemical measurements were performed with a μSTAT 400 (Metrohm DropSens, Oviedo, Spain) potentiostat/galvanostat and Autolab PGSTAT302 N equipped with the FRA32M module (Metrohm Autolab b.v., Utrecht, The Netherlands) at ambient temperature in a three-electrode working cell. A glassy carbon electrode (GCE, 2 mm in diameter, OhmLiberScience, Saint-Petersburg, Russia) was used as a working electrode and transducer in biosensor assembling. The Ag/AgCl/3.0 M NaCl reference electrode (ALS Co., Ltd., Tokyo, Japan, Cat. No. 012167) was used in voltametric measurements and Ag/AgCl/3.0 M KCl (Metrohm Autolab b.v. Cat No. 6.0733.100) in impedimetric measurements. Pt wire (ALS Co., Ltd., Cat. No. 002233, or Metrohm Autolab b.v., Cat. No. 6.1248.000) was applied as a counter electrode.

Cyclic voltammetry was chosen for the study of the DNA–polyphenothiazine composite formation and DNA–doxorubicin interaction. This method has a well-developed theory, an intuitively understandable explanation of the peak changes on voltammograms, and it is compatible with portable biosensors’ requirements in the framework of point-of-care testing (POCT). Here, 25.5 cycles were used for electropolymerization assuming finalizing the synthesis at the highest anodic potential. All the measurements were performed with five individual sensors. Calculation of the peak currents was performed using the NOVA Software (Metrohm Autolab b.v.) after baseline correction. Geometric electrode area was used in calculation of the current densities.

Electrochemical impedance spectra (EIS) were recorded at the equilibrium potential in the frequency range from 100 kHz to 0.04 Hz, with 50 frequencies in the scan, at an amplitude of 5 mV. Equilibrium potential was determined as a half-sum of the peak potentials recorded in the equimolar mixture of K_3_[Fe(CN)_6_] and K_4_[Fe(CN)_6_]. The impedance parameters were determined by fitting data with the Randles equivalent circuit [*R_s_*(*Q*[*R_ct_W*])], where *R_s_* represents the resistance of electrode material, of all electric contacts, and the uncompensated ohmic resistance of solution, *Q* is the constant-phase element (CPE) representing the non-ideal capacitive behavior of the electrical double-layer; *R_ct_* is the charge transfer resistance (interfacial electron transfer), and *W* the Warburg element (a virtual electronic component that models the diffusion of electroactive species). Equivalent circuit fitting was performed with the NOVA software (Metrohm Autolab b.v.). 

Scanning electron microscopy (SEM) images of the electrode coatings were obtained with the high-resolution field emission scanning electron microscope Merlin™ (Carl Zeiss, Jena, Germany). ZeissSmartSEM software was used for image processing.

Statistical data treatment was performed using OriginPro 8.1 software (OriginLab Corporation, Northampton, MA, USA).

### 2.2. Electrode Modification

Before use, the GCE was mechanically cleaned with silicon carbide abrasive paper (P5000) and washed with deionized water and ethanol. After that, it was electrochemically activated in 0.2 M sulfuric acid containing 50 vol.% acetone by cycling the potential until stabilization of the voltammogram. Then, an aliquot of the PhTz dissolved in the acetone was added to its final concentration of 72 μM. The electropolymerization was performed by multiple cycling of the potential in the range from −0.5 to 1.3 V, scan rate of 0.1 V/s. The DNA immobilization was performed by two various approaches, i.e., by addition of the DNA aliquot to the working solution on the electropolymerization step or by drop casting of the DNA solution onto the polyPhTz/GCE surface followed by drying the electrode on air at ambient temperature and its rinsing with deionized water. Both methods differ in the distribution of the DNA molecules in the self-association products on the electrode interface and in accessibility of the DNA molecules for the intercalator (doxorubicin as model analyte).

## 3. Results and Discussion

### 3.1. PhTz Electropolymerization

**Figure 2:**
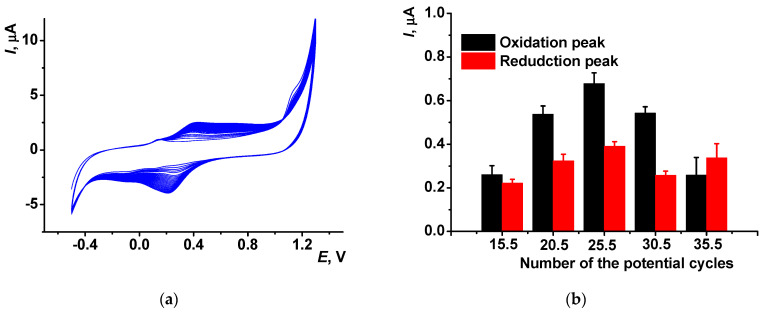
(**a**) Cyclic voltammograms of 72 µM PhTz dissolved in H_2_SO_4–_acetone (1:1 *v*/*v*) mixture, 100 mV/s; (**b**) The dependence of the peak currents recorded on the modified GCE in 0.1 M HEPES + 0.1 M NaNO_3_ in the absence of PhTz monomer on the number of the potential cycles. Average ± S.D. for five individual sensors.

The presence of both phenyl imine and phenothiazine fragments in the PhTz structure is interesting from the point of view of the electropolymerization because they can alter the redox activity and an electron–ion conductivity depending on the centers involved in the formation of the polymeric product. Figure 2a represents cyclic voltammograms recorded in the H_2_SO_4_–acetone (1:1 *v*/*v*) solution containing PhTz.

The irreversible anodic peak at 1.1 V corresponds to the formation of the radical initiating the polymerization. If the maximal potential was chosen below this value, no significant changes were observed on multiple cycling. Similar activation of the polymerization at high anodic potential was reported for the electropolymerization of aniline and phenothiazine dyes [47,56]. Increasing the number of the cycles of the potential between −0.5 and 1.3 V consecutively increased the peaks attributed to the products of electrosynthesis (0.4 V on anodic branch of the voltammogram and 0.2 V at the cathodic branch). These changes were observed until the 25^th^ cycle, after which the currents started decreasing due to blocking the GCE surface with the polymerization products, which complicated access of the monomers to the electrode interface. Such non-regular changes in the peak currents are typical for the electropolymerization of the phenothiazine and phenoxazine derivatives [58,59]. The dependence of the oxidation/reduction peak currents on the number of electropolymerization cycles is presented in Figure 2b.

In the following experiments, 25.5 cycles of electropolymerization were used. A half-cycle corresponds to finishing the potential scan at the highest anodic potential required for the accumulation of the maximally oxidized product able to electrostatically interact with negatively charged DNA molecules.

**Scheme 1:**
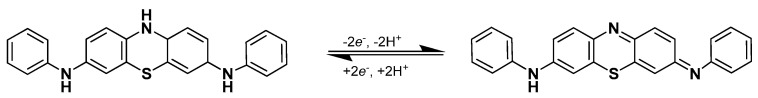
Redox equilibrium of the monomeric form (PhTz) and the fragment of the polymer (polyPhTz) responsible for the first peak pair on voltamogram.

The redox activity of the product of electropolymerization studied in HEPEs solution with no monomer corresponded to the reversible reduction/oxidation of the phenothiazine core, as shown in Scheme 1, for a single unit of the polymer present in a neutral (non-protonated) form. The formation of linear polymers for aminated phenothiazine derivatives was recently proved by ATR FTIR based on the signals related to phenylene diamine and quinone fragments [60].

**Figure 3:**
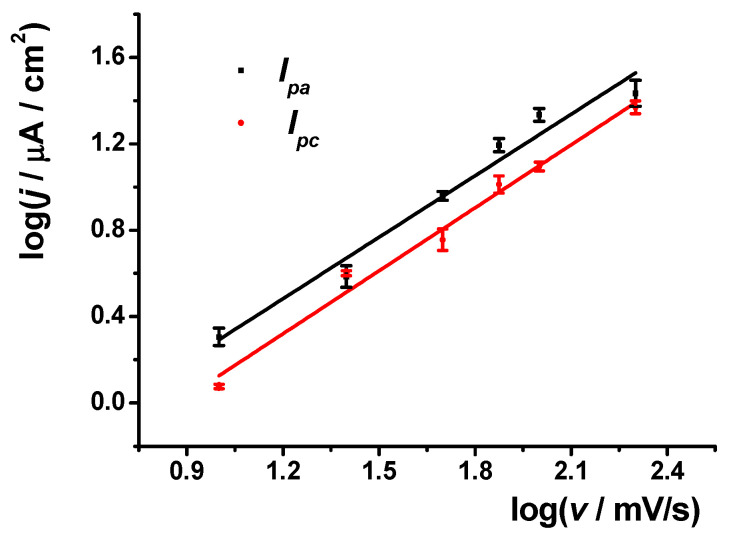
The dependence of the polyPhTz peak potentials on the scan rate. Average ± S.D. for five individual sensors.

The dependence of the peak currents related to the PhTz redox conversion on the scan rate was assessed in 0.1 M HEPES. The slopes of plots in bi-logarithmic coordinates were near 1, indicating a surface-confined limiting step of the electron transfer (Figure 3). 

Appropriate equations are presented in the following Equations (1) and (2):

log(*j_pa_*, μA/cm^2^) = −(0.66 ± 0.14) + (0.95 ± 0.08) × log(ν, mV/S), *R^2^* = 0.965 (1)

log(*j_pc_*, μA/cm^2^) = −(0.85 ± 0.10) + (0.97 ± 0.06) × log(ν, mV/s), *R^2^* = 0.986 (2)

where *j_pa_*, *j_pc_* are the peak current densities, µA/cm^2^, of the anodic and cathodic peaks, respectively, and ν is the scan rate, mV/s. When electropolymerized in neutral media, polyPhTz showed mixed adsorption–diffusion control of the electron transfer [53].

**Figure 4:**
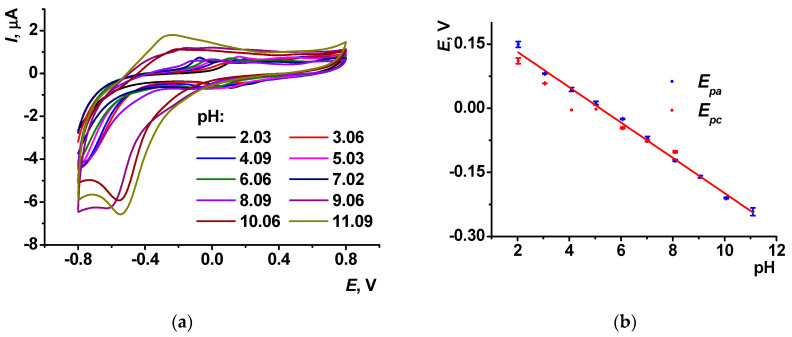
(**a**) Cyclic voltammograms recorded on the GCE covered with polyPhTz in 0.1 M HEPES + 0.1 M NaNO_3_, 100 mV/s, at various pH; (**b**) the dependence of the peak potentials of anodic (*E_pa_*) and cathodic (*E_pc_*) peak potentials on the pH. Average ± S.D. for five individual sensors.

The pH dependence of the peaks is shown in Figure 4a. In acidic media, the peak currents are similar to each other, and the peak potentials regularly shifted with the pH to their lower values (Figure 4b). The cathodic peaks overlapped the peaks of oxygen reduction so that maximal pH available was limited to 8.0. Within the range from pH = 2.0 to 8.0, the slope of the dependence (55 mV/pH for cathodic and 41 mV/pH for anodic peaks) corresponded to the transfer of equal number of electrons and hydrogen ions.

### 3.2. DNA Immobilization

#### 3.2.1. Voltammetric Study

If DNA was added to the reaction media in the electropolymerization step, appropriate sensors are denoted as GCE/(polyPhTz + DNA). For them, implementation of DNA is promoted by electrostatic interactions between positively charged PhTz and negatively charged phosphate residues of the DNA helix.

**Figure 5:**
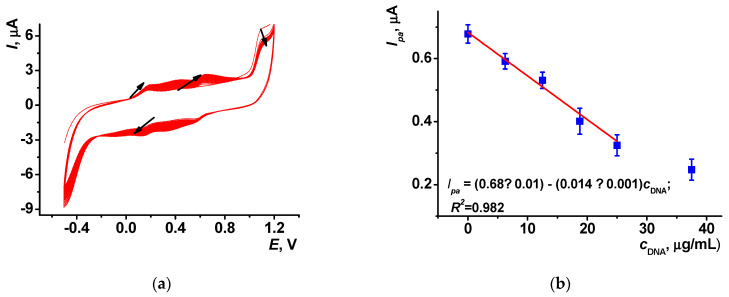
(**a**) Multiple cyclic voltammograms recorded on GCE in a solution of 72 µM PhTz and 12.5 µM DNA1. Arrows show the direction of the changes with an increasing number of the cycles; (**b**) the dependence of the anodic peak current of the GCE modified with polyPhTz/DNA1 on the concentration of DNA in the solution for electropolymerization. Average ± S.D. for five individual sensors.

As an example, Figure 5a illustrates electropolymerization performed in the presence of 12.5 µg/mL DNA1 from salmon sperm. The addition of DNA1 increased the currents on cyclic voltammograms against those shown in Figure 2a, where no DNA was added. In the course of electropolymerization, changes in the peak pair attributed to the monomeric PhTz at about 0.2–0.4 V are supported with by the appearance of a comparable in heigh anodic peak at 0.6 V, which can be due to the redox activity of the polyPhTz. The DNA’s influence increases with its quantities and is especially obvious in the range from 12.5 to 18.7 µg/mL (Figure 5b). To reach a maximally accurate signal, 12.5 µg/mL DNA addition was used in the following experiments. 

Incubation of the GCE modified with polyPhTz in the DNA solution resulted in its electrostatic accumulation on the polymer surface. The appropriate DNA sensor is denoted as GCE/polyPhTz/DNA. In this protocol, 10 µL aliquot of 0.1 µg/mL DNA was drop-casted on the electrode and left for a certain time period for the adsorption of the biopolymer. It was established in preliminary experiments that 20 min incubation was sufficient to reach a stable reproducible response toward DNA. Being simpler than previously described DNA entrapment methods into the growing electrosynthesized film, this protocol can be complicated by leaching adsorbed DNA from the surface layer. It was checked by monitoring the signal by alternating the incubation of the sensor in deionized water and HEPES buffer (Figure S1). The deviation in the peak currents for six repeated cycles measurement washing (measurement-to-measurement repeatability) was about 2.3%, and for six individual sensors (sensor-to-sensor repeatability) it was 3.3%. Similar experiments with the GCE/polyPhTz containing no DNA showed a small but regular decrease in the polyPhTz peak currents, probably due to partial desorption of the soluble products of oligomerization. Thus, DNA adsorption stabilized the surface film obtained. A further increase in the incubation period to 30–60 min, as well as higher DNA loading (up to 10 mg/mL in 10 μL casted per electrode) decreased the peaks on the resulting voltammograms (Figure S2). These changes are hardly attributed to the leakage of the surface components and are rather related to recharging the electrode interface and/or lower reversibility of the PhTz redox conversion caused by increased quantities of the highly charged DNA molecules transferred.

**Figure 6:**
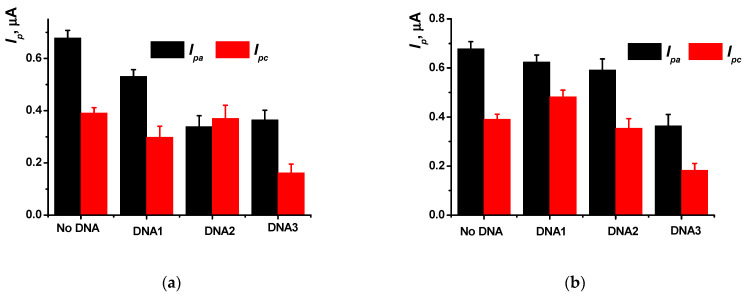
Influence of the DNA source on the PhTz peak currents: (**a**) GCE/(polyPhTz + DNA) sensor; (**b**) GCE/polyPhTz/DNA sensor. Average ± S.D. for five individual sensors.

The DNA source affected the polyPhTz currents both in the case of GCE/(polyPhTz + DNA) and GCE/polyPhTz/DNA sensors (Figure 6). We have not found any evidence of DNA damage or denaturation during the electrolysis in acidic media for all the DNA sources. Probably, this was due to a short electrolysis time and electrostatic stabilization of the native DNA structure by depositing polyPhTz molecules.

In both protocols, the maximal changes in the peak currents were obtained with the application of high-molecular DNA3 from chicken erythrocytes. This might result from lower solubility of the poly(PhTz)–DNA3 complexes and from the accumulation of a larger negative charge affecting the stability of the coating. The difference in the appropriate peak currents was higher for the layered coating (GCE/polyPhTz/DNA sensor). DNA1 and DNA3 caused changes by about 50%, while those for the DNA implemented in the growing polymer film (GCE/(polyPhTz + DNA) were about 30%. This indirectly provides evidence in favor of partial coverage of the electrode with non-conductive DNA molecules. The above parameter is sensitive to the specific square of the electrode occupied with DNA molecules. Regarding DNA1 and DNA2, their implementation into the polymer film mostly affected oxidation peaks, whereas DNA deposition onto the electrode moderately influenced reduction peaks, too. This might result from the molar mass distribution of both DNA samples. DNA2 from fish sperm contains a larger variety of double-stranded and single-stranded DNA fragments [60] that can more effectively block the access to the redox active sites on the electrode surface in comparison with DNA1 of a more regular structure. Difference in the DNA accumulation is mostly obvious on the reverse branch of voltammograms, where the product of primary electron transfer is re-reduced and the coordination of oppositely charged functional groups of the layer interface affects the shape of cyclic voltammogram to a higher extent.

The electrochemical behavior of the PhTz was compared with that of the derivatives containing additional charged groups at the phenothiazine core, i.e., the diaminated and dicarboxylated derivatives studied in [59]. All the compounds mentioned showed the ability of electropolymerization, but the quantities of the products deposited and the pH dependence of redox activity depended on the number and charge of terminal groups. The introduction of carboxylic groups suppressed the accumulation of polymeric products against aminated derivatives. Diaminated derivatives demonstrated more complicated voltammograms with the peaks of monomeric and polymeric forms comparable in height. Structurally relative thionine and azure dyes exerted the activity in electropolymerization sensitive to the steric loading of the amino groups [29,32,33,37,58]. Cyclic voltammograms of Azure A and Azure B demonstrated similar broad peaks of the polymer accumulation but the quantities of appropriate products assessed from the currents were significantly lower. In all cases mentioned, the electropolymerization allowed for DNA deposition. The thinner the electropolymerized film, the higher the sensitivity of the voltametric parameters to the DNA intercalators was. This might be explained by compatibility of the charge distribution in positively charged polymer and DNA molecules expected for thin films and to some extent by lower solubility of the polymerized dyes providing their dense contact with the underlying electrode surface. The possibility of the PhTz electropolymerization on bare GCE with no additional modifiers is another advantage of the sensor developed versus the assemblies based on pre-concentration of the monomeric precursors on carbon black. The influence of amino groups of the PhTz molecule on the reversibility of the redox reactions estimated by ferricyanide redox probe was also lower than that of the phenothiazines with amino groups directly attached to the phenothiazine core. This partially compensates for exhausting pH sensitivity of the response mentioned for the analogs studied. Similar trends were discussed in the application of phenothiazine dyes for mediation of the oxidation of small molecules (see reviews [31,36,58]).

#### 3.2.2. EIS Study

**Figure 7:**
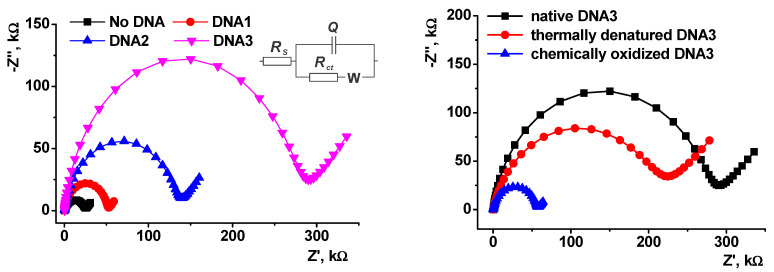
The Nyquist diagrams obtained with GCE covered with polyPhTz and DNA from various sources. Inset: equivalent circuit and semi-circle area of the diagram).

The DNA accumulation onto the polyPhTZ layer was then studied by the EIS with 0.01 M [Fe(CN)_6_]^3−/4−^ as the redox probe. The redox probe concentration was specified to reach well-resolved peak currents on the modified electrodes. The Nyquist diagrams obtained with the GCE modified with DNA from various sources are presented in Figure 7. The EIS parameters obtained by fitting the experimental data with a [*R_s_*(*Q*[*R_ct_*W])] equivalent circuit (see the description of variables in the Section 2.1 Apparatus) are presented in Table 1. The Randles equivalent circuit used reflects the electrochemical phenomena on the electrode interface in terms of charge distribution at the electric double layer and kinetics of electron exchange with the redox probe.

The semi-circle area on the Nyquist diagram corresponds to the kinetic control of the electron exchange whereas the linear piece in the range of low frequencies corresponds to the diffusion control of the reaction. The depressed form of the semi-circles is caused by non-ideal behavior of the CPE and coincides well with the roughness factor *N*, which differs from 1 corresponded to capacitance behavior. Such a behavior can be explained by uneven distribution of the modifiers along the electrode, or by high roughness and porosity of the surface layer on the electrode [61]. With the increased average molar mass of the DNA (from DNA1 to DNA3), the charge transfer resistance *R_ct_* increased because of the accumulation of a higher negative charge and repulsion of anionic ferro- and ferricyanide ions of the redox probe on the electrode interface. A sharp decrease in the *Q* value observed for DNA3 with maximal molar mass is due to the same reason. The EIS data agrees with the voltammetry studies of the surface layer assembling and with the conclusion on the importance of negative charge of DNA helix and its alteration after the DNA deposition (see Figure 6b) with minimal peak currents recorded for DNA3 loading.

It is interesting that the redox signals of the DNA sensors were sensitive to thermally denatured and chemically oxidized DNA. Such a differentiation has already been described for similar DNA sensors [29,32,33,62] and was referred to changes in the flexibility of the DNA molecules. High-molecular DNA3 was heated for 30 min to 95 °C and then sharply cooled for 5 min in the ice bath prior to its introduction in the surface layer of the GCE/polyPhTz/DNA sensor. This resulted in partial unwinding of the DNA strands and primary structure distortion (formation of abasic sites etc.) [63]. The EIS experiment showed the decrease in the *R_ct_* value to 212 ± 8 kΩ, indicating satisfactory better electroconductivity of the polymer layer [64].

#### 3.2.3. SEM Study

**Figure 8:**
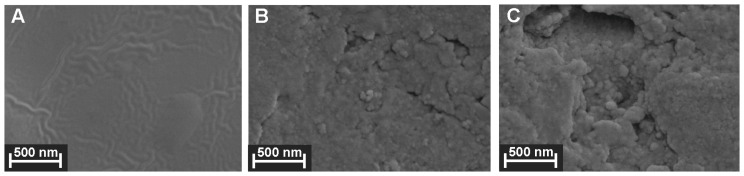
SEM images of GCE covered with electropolymerized PhTz (**A**) (25.5 cycles, 72 μM PhTz) and image obtained with DNA1 added to the solution (37.5 μg/mL) (**B**) or drop-casted on the polymer film (10 μL of 100 ng/mL per electrode) (**C**).

The assembling of the polyPhTz/DNA surface layer was proved by SEM. On bare GCE, electropolymerization resulted in the formation of a dense film with a wavy surface (Figure 8A). The addition of DNA either to the PhTz solution or on the surface of the polymer film resulted in the formation of the structured elements with microcrystalline inclusions and cavities randomly distributed on the surface (Figure 8B,C). The morphology of the polyPhTz/DNA1 layer was similar for both protocols of the DNA introduction but the depth of the cavities and probably the thickness of the layer were bigger for the DNA deposited on the film. One could see the roundish particles within the surface layer, mostly obvious for the side walls of the cavities and after the DNA implementation (Figure 8C). Their size was in between 40–80 nm and tended to increase with the DNA molar mass. 

### 3.3. Measurement Conditions

#### 3.3.1. Cyclic Voltammetry

The dependence of the PhTz peak currents on the DNA loading and electrostatic interactions within the layer as a driving force of the changes made it possible to propose the DNA sensor for the determination of low-molecular species able to specific interaction with DNA. In this work, doxorubicin was used as a kind of model intercalator because of the importance of its sensitive detection in biological fluids and well-elaborated mechanism of interaction with DNA. 

**Figure 9:**
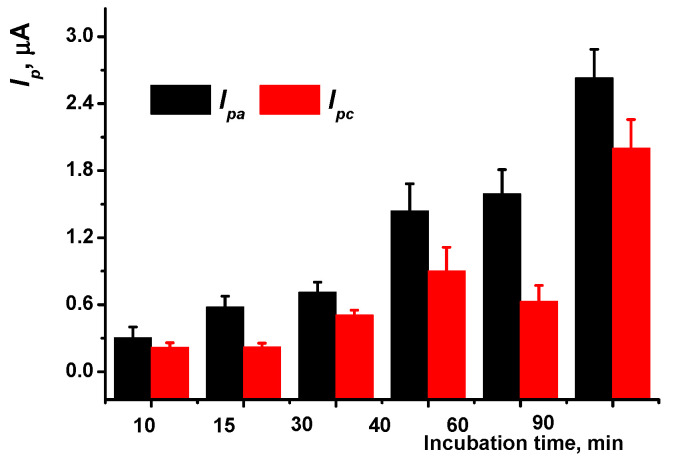
The polyPhTz peak currents recorded after incubation of the GCE/polyPhTz/DNA1 sensor in 0.1 mM doxorubicin solution for 10–90 min. Average ± S.D. for five individual sensors.

Indeed, incubation of the GCE/polyPhTz/DNA sensor in the doxorubicin solution resulted in an increase in the peak currents to the values obtained for the polyPhTz coating with no DNA (Figure 9 for DNA1, the oxidation peak current for the GCE/polyPhTz was equal to 0.62 μA). 

Intercalation of the drug in the DNA helix results in an increase in the biopolymer specific volume, partial unwinding, and spatial separation of the negative charges of the phosphate residues of the DNA backbone. These changes promote transfer of the phenothiazine units in oxidized (positively charged) form and hence increase their currents on voltammogram. The influence of the drug on the DNA sensor signals was rather stable within the 10–30 min incubation period in the doxorubicin solution. A further increase in the incubation period resulted in a lower reproducibility of the response, compensated for by higher currents.

**Figure 10:**
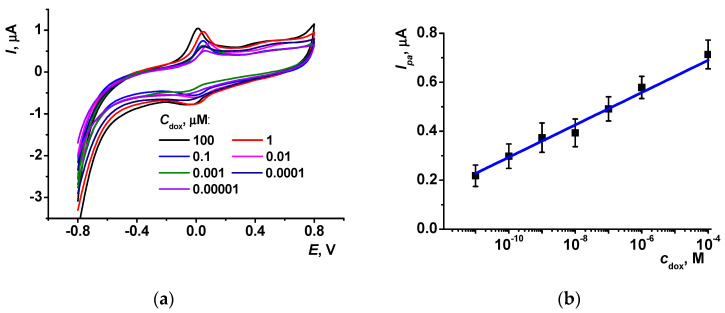
Influence of the doxorubicin on the PhTz peak currents: (**a**) cyclic voltammograms recorded after 15 min incubation with the GCE/polyPhTz/DNA1 sensor; (**b**) calibration curve of doxorubicin. Average ± S.D. for five individual sensors.

Changes in the oxidation peak currents caused by doxorubicin were more reproducible than those of cathodic peaks and they were used for the quantitative analysis of the drug. Figure 10 shows changes in the voltammograms for 15 min incubation of the GCE/polyPhTz/DNA1 sensor in the doxorubicin solution. The results obtained with DNA2 and DNA3 are shown in Figure S3.

The analytical characteristics of the doxorubicin determination are summarized in Table 2. The limit of detection (LOD) of doxorubicin was assessed with S/N = 3 criterion. As one can see, application of DNA from salmon testes showed the broadest range of concentrations determined. The use of DNA2 from fish sperm was found to be less effective. The slope of the linear part of the plot was similar that that obtained with DNA1, but the interval was narrower and the relative deviation of the peak current was higher. This might result from the rather high content of single-stranded DNA fragments. They do not interact with doxorubicin in accordance with the intercalation mechanism. High-molecular DNA3 from chicken erythrocytes was less sensitive to the contact with doxorubicin, probably due to a much larger number of binding sites and the higher stability of less soluble polyelectrolyte complexes on the electrode interface. 

The results obtained are comparable or better than those reported for other electrochemical sensors (Table S1). It should be noted that doxorubicin itself is electrochemically active and shows its own peaks on voltammograms but at much higher concentrations so that they do not interfere with the redox signals of the polyPhTz layer.

The use of redox active polymers offers sub-nanomolar detection limits sufficient for the drug residues determination in urine and blood serum. The common concentrations of doxorubicin reported for patients with gastrointestinal cancer were on the level of 10 nM within 60–100 min after the drug administration [65]. In plasma of superficial bladder cancer patients, doxorubicin was detected at a maximal level of 0.9–8 nM (mean 2 nM for six weekly treatments of 40 mg doxorubicin in 20 mL physiological saline [66]). The concentration of doxorubicin in urea depends on the dose and excretion period. Within two weeks, about 50% of the drug is released with urine. Its stability depends on the pH and other conditions but can be assessed as 0.1–10 µM [67,68]. Thus, the comparison of the DNA sensor performance with the drug levels makes it possible to conclude that possible interference expected from the matrix components can be easily eliminated by the sample dilution. 

The only DNA sensor showing lower detectable concentrations contained layered polyaniline and DNA layers (polyaniline–DNA–polyaniline). Polyaniline offered higher electrostatic attraction of DNA than polyPhTz and is more sensitive to DNA-specific interactions but had limitations in terms of pH working region and stability in neutral media. Electrochemical sensors mostly utilize mediated oxidation of doxorubicin and/or its preliminary accumulation in the surface layer. High working potential prevents their application in complex media containing oxidizable species (drug stabilizers, food additives) though the stability of the response in protein-containing media is rather high. It should be noted that the approach to the layered deposition of polyPhTz utilized in this work offers a very simple and reproducible protocol of sensor assembling and makes it possible to obtain inexpensive but reliable sensors for single use in medical diagnostics, pharmaceutics, chemotherapy monitoring, and other purposes related to oncology and antitumor drug design.

#### 3.3.2. Measurement Precision and Sensor Lifetime

Sensor-to-sensor repeatability was assessed using a set of five electrodes modified with the same reagents. The R.S.D. was found to be 5.6% (0.1 nM doxorubicin, 20 min incubation) and the period of 25% decay of the signal was equal to 15 days (Figure S4a). In dry conditions, polyPhTz retains its electrochemical activity for at least 60 days with no DNA and 90 days when covered with 0.1 mg/mL DNA (Figure S4b). The R.S.D. of the signal toward 0.1 nM doxorubicin increased to 9% at the end of the storage period. In working solution, the reliable detection of doxorubicin (R.S.D. < 10%) was achieved within three days. 

#### 3.3.3. Selectivity and Real Sample Assay

The possibility of direct determination of doxorubicin in serum is important due to the high risks of cardiotoxicity and individual variability in the pharmacokinetics of the drug. We have checked the signals of other antitumor drugs, including the same anthracycline core as well as commercial medications with different stabilizers and common components of the fluids to be sure that the complex character of the samples did not interfere with the doxorubicin determination. 

Determination of doxorubicin is based on the general mechanism of intercalation, which is common for all the anthracycline preparations. To compare the influence of other representatives of this group of drugs, the concentrations exerting a 15% shift in the signal (IC_15_) were determined with the GCE/polyPhTz/DNA1 sensor. They were equal to 5 pM for doxorubicin, 20 pM for daunorubicin, 30 pM for idarubicin, and 18 nM for valrubicin for GCE/polyPhTz/DNA1 sensor, at 15 min incubation. A 15% shift was chosen assuming a 5% deviation of the signal typical for electrochemical sensors. Relative changes in the anthracycline detection coincide with the previously reported results obtained with other electrochemical DNA sensors. Thus, the LODs of 0.01 nM doxorubicin, 0.1 nM daunorubicin, and 0.2 nM idarubicin were obtained using the EIS technique with the DNA sensors based on thin polyaniline films [69]. Valrubicin showed lower sensitivity with DNA sensors with carbon nanomaterials and Au nanoparticles (LOD 18 nM [70]). It should be noted that anthracycline drugs are mostly applied separately, and medications differ in the nature of auxiliary components providing target delivery of the substances to the solid tumor (lipids, stabilizers, etc.). Regarding other drugs, IC_15_ = 0.01 mM was obtained for sulfamethoxazole. Cyclophosphamide, prednisone, and dacarbazine, commonly applied together with doxorubicin in chemotherapy regimens, showed an insignificant irregular influence on the GCE/polyPhTz/DNA1 signal at concentrations exceeding 1.0 mM. 

**Figure 11:**
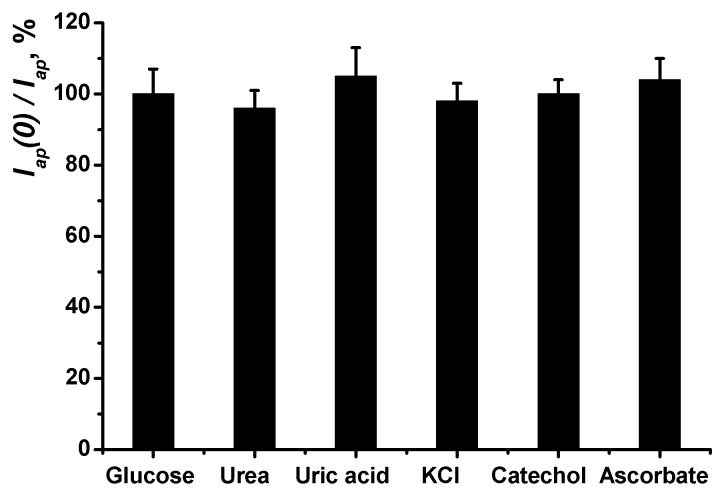
Relative changes in the polyPhTz anodic peak current recorded with the GCE/polyPhTz/DNA1 sensor after 15 min incubation in 1.0 nM doxorubicin solution (*I_pa_*(0)) and in the presence of 1.0 mM interferences (*I_pa_*). Measurements in 0.1 M HEPES + 0.1 M NaNO_3_, 100 mV/s. Average ± S.D. for five individual sensors.

Some matrix components capable of redox conversion on the electrode can also interfere with the doxorubicin signal. In Figure 11, the relative changes in the signal toward 1.0 nM doxorubicin are compared with those obtained in the presence of 1.0 mM interferences (glucose, uric acid, catechol, ascorbate). Furthermore, urea and KCl as macro components of urine were tested in the same conditions. Here, 100% corresponded to no interference, and the higher is the difference from 100%, the bigger the undesirable contribution of the additives. One can see that the effect is below the standard deviation of the signal for all the model compounds tested.

The influence of serum proteins was modeled by bovine serum albumin added to the HEPES buffer. Moderate level of albumin typical for the adults’ blood serum was selected for model experiments (41.4 mg/mL).

Furthermore, Ringer–Locke’s solution (0.45 g NaCl, 0.021 g KCl, 0.016 g CaCl_2_·2H_2_O, 0.005 g NaHCO_3_, 0.015 g of MgSO_4_, and 0.025 g of NaH_2_PO_4_·2H_2_O per 50 mL of water [71]) was used to estimate the influence of plasma electrolytes. The recoveries of 105 ± 10% and 105 ± 8% were found for 0.1 nM doxorubicin in both cases. 

Doxorubicin-TEVA^®^ and Doxorubicin-LANS^®^ were dissolved in 0.1 M HEPES buffer and applied for the incubation as described above for a standard doxorubicin solution. The recovery was calculated from the calibration plot obtained in HEPES buffer, pH = 7.0. Three nominal concentrations of doxorubicin (10 and 100 pM, and 1.0 nM) were tested, and average recovery was equal to 90 ± 10% (Doxorubicin-TEVA) and 95 ± 10% (Doxorubicin-LANS). Thus, stabilizers present in the medications (lactose and mannitol) did not interfere with the doxorubicin determination. 

## 4. Concluding Remarks

Fast and reliable determination of anthracycline drugs in biological fluids is demanded in two areas related to the anticancer drugs’ administration and to the selection of new drugs with reduced toxic effects. The rather high cardiotoxicity of doxorubicin and its analogs, together with hepatoxicity, limit their application and increase health risks in the population. Although they are effective, especially in the solid tumor family, anthracycline drugs need monitoring for an effective dose depending on metabolism specificity and other influencing factors. DNA-based biosensors represent an approach to the quantification of DNA–drug interactions because they utilize biological targets, i.e., native DNA molecules, as both recognition elements and signal forming components. Common approaches based on direct analysis of accumulated drugs or detection of the products of DNA damage are rather sensitive but require sophisticated equipment and skilled staff. Electrochemical DNA sensors can offer attractive opportunities, e.g., very simple assembling and signal measurement protocol, and intuitively understandable interpretation of the results. 

Electropolymerization is one of modern trends broadly applied for assembling biorecognition layers of biosensors. Regarding DNA–drug interaction, this protocol allows self-assembling of the surface layer consisting of oppositely charged counterparts (polymers and DNA molecules), the electrochemical properties of which depend on both the drug nature and conditions of their interaction. The electropolymerization protocol is easily modified for particular tasks and can control the growth and assembling of the surface film. Furthermore, the redox properties of the nanoaggregates formed are sensitive to the DNA structure and intercalation of the anthracycline drugs. One-pot synthesis and the possibility of entrapping DNA in the polymer film via weak multipoint interactions are the advantages of the approach over the use of conventional modifiers, e.g., carbon nanoparticles or quantum dots. The low cost of consumables and rather high reproducibility of main biosensor parameters should also be mentioned. 

However, the advantages of electropolymerization can be applied only in the case of careful control of the assembling conditions and specifying the factors that can influence the sensitivity of drug detection. Variation in the monomer structure and implementation of charged terminal groups near the redox centers makes it possible to specify the main requirements to the assembling of the biorecognition layer for particular analytes and real sample assays.

In this work, a new derivative of phenothiazine has been investigated for the first time in the conditions of polymerization in acidic conditions. The choice of the phenothiazine monomer was governed by the existence of two alternative ways of polymerization that would result in the polymeric films with various distribution of charge and ability to interact with DNA. In basic media, phenothiazine-like polymerization takes place, as was shown for similar structures of phenothiazine dyes [48,54]. In acidic media, electropolymerization via aniline fragment can take place, resulting in the formation of an electroconductive coating, such as polyaniline. We have found that multiple cycling the potential in the monomer solution performed in sulfuric acid mixed with an equal volume of acetone resulted in the deposition of a stable film easily adsorbing DNA both on the surface and by entrapment from the reaction media. The polymers obtained from acidic media exert higher sensitivity toward the DNA intercalator and lower deviation in the signal in repeating records of cyclic voltammograms than similar DNA-sensors assembled in a neutral solution. 

The implementation of DNA was proved by EIS data and the dependency of the redox activity of the coating on the DNA source and biopolymer deposition protocol. Indeed, the influence of DNA is based on two simultaneous reactions, i.e., the limitation of electron transfer by partial blocking of the electrode surface and the promotion of the formation of oxidized (positively charged) polymers due to electrostatic interactions with the phosphate residues of the DNA backbone. The balance between these two processes makes the recorded redox peaks on voltammograms sensitive to the DNA source and interaction with model intercalator (doxorubicin). SEM images confirmed the nanostructuring of the surface film because of the uneven distribution of the polyelectrolyte molecules and different distances between the charge locations. The roughness of the coating promotes the DNA implementation and increases the active surface area. The latter one positively affects the amperometric signal and redox conversion of the polymer fragments responsible for drug detection.

Three different DNA preparations were used. High-molecular DNA from chicken erythrocytes formed the most stable complexes with polyPhTz. However, the response of the appropriate biosensor toward doxorubicin was lower than that for the biosensors implementing DNA from other sources. DNA from fish sperm with maximal variety of the molar mass and rather high content of single-stranded DNA strands was found to be less applicable in the DNA sensor assembly, both from the point of view of the redox characteristics of the surface layer and its sensitivity toward the intercalator. 

Regarding the DNA implementation, both protocols suggested (addition of DNA to the reaction media and its adsorption onto the polymer layer after electropolymerization) showed similar effects on the electrochemical characteristics of the composites formed. This confirms the idea of the predominant influence of charge separation on the redox equilibrium of polyPhTz. Meanwhile, the influence of doxorubicin was higher when DNA was placed onto the polymer film. This can result from better accessibility of the DNA active sites for the drug molecules and/or from the difference in the roughness of the layer indirectly shown by SEM. On the other hand, DNA adsorption complicates the protocol of the biosensor assembling and slightly decreases the accuracy of the signal measurement.

Intercalation of doxorubicin resulted in partial unwinding of double-stranded DNA and changes in the negative charge separation and was found to be selective against sulfanilamide and some drugs applied together with anthracyclines. Relative changes in the sensitivity toward anthracycline preparations tested were similar to those already described in the literature for similar DNA sensors [29,69,70]. Common components of biological fluids, stabilizers of commercial medications, and drugs commonly applied together with doxorubicin in chemotherapy regimens (cyclophosphamide, prednisone, and dacarbazine) did not regularly affect the doxorubicin determination. 

Polyphenothiazine derivatives show some advantages over other electropolymerized materials used in biosensors. Their redox activity is less pH sensitive and can be monitored both in acidic (like polyaniline) and neutral media, including physiological pH range. Then, the high density of the positive charge of oxidized forms promoted self-assembling with polyanilines including DNA molecules. Lower sensitivity against three-layered polyaniline–DNA–polyaniline biosensors can be explained by the application of diffusionally free mediator, methylene blue, that was accumulated in the DNA and substituted with doxorubicin molecules during the incubation step. For this reason, such a biosensor cannot be named as reagent free and needs more steps for its manufacture and signal measurement. Other DNA sensors described for doxorubicin determination exploit nanomaterials that are synthesized and implemented in the sensing layer by several steps complicating the biosensor operation. Single-walled carbon nanotubes and Ag and Pt nanoparticles improve the conditions of electron exchange and increase the specific electrode surface accessible for electropolymerized monomers and DNA molecules. The function of TiO_2_ and MgO is less understandable. They do not exert redox activity and are non-conductive. Probably, they adsorb species involved in the electrode reactions and increase their local concentration on the electrode. Whatever the case, the more complicated the layer, the more steps there are in its assembling and the more sources of measurement deviations.

High sensitivity of the doxorubicin determination observed for the DNA sensor developed can be attributed to a combination of various factors, i.e., accessibility of DNA in the nanocomposite with a polymeric form of dye for low-molecular intercalators, a well-developed surface of the film accumulating both the DNA and drug molecules, and high quantities of DNA implemented in the surface layer.

Nano- and picomolar detectable concentrations made it possible to avoid the interference of serum proteins and plasma electrolytes and to monitor typical levels of the anthracycline drugs currently used in cancer therapy. 

The progress in the development of such DNA sensors assumes further efforts in establishing optimal structure of phenothiazine derivatives as the redox polymer precursors and improvement of metrological characteristics of the signals, especially during the storage period. The electrochemical DNA sensor developed can be applied for monitoring drug release in chemotherapy and screening new antitumor drugs able to intercalate DNA. Furthermore, the electropolymerization protocol elaborated can be useful for preparing organic films suitable for optical of sensing organic species using nanoplasmonic approaches and metasurfaces, artificial 2D materials with peculiar electromagnetic properties. Electropolymerized films can serve as photosensitizers in the catalytic reaction of the oxidation of organic matter and as components of electrochromic devices. Although such an application is beyond the topic of this investigation, the electrochemical characterization of the polymeric films formed can stimulate their application in the above areas.

## Figures and Tables

**Table 1:** EIS data of GCE modified with polyPhTz and drop-casted DNA from various source. Average ± S.D. for five sensors.

Modifier	*R_S_*, Ω	*R_ct_*, kΩ	*Q*, µF	*N*
PolyPhTz	162 ± 8	24.5 ± 3	0.27 ± 0.04	0.86
PolyPhTz/DNA1	191 ± 8	51.2 ± 4	0.32 ± 0.05	0.91
PolyPhTz/DNA2	171 ± 10	134 ± 6	0.08 ± 0.03	0.88
PolyPhTz/DNA3	172 ± 12	277 ± 6	0.04 ± 0.01	0.90

**Table 2:** Analytical characteristics of doxorubicin determination with GCE/polyPhTz/DNA, *I_pa_*, μA = a + b × (*c*_dox_, M). Average ± S.D. for five sensors, where *n* is the number of experimental points within the linear range of concentrations determined.

DNA Source	*a* ± Δa	*b* ± Δb	*R* ^2^	*n*	Linear Concentration Range	LOD
DNA1	0.99 ± 0.02	0.070 ±0.003	0.9898	7	10 pM–0.2 mM	5 pM
DNA2	1.12 ± 0.11	0.078 ± 0.012	0.9784	5	20 pM–12 nM	10 pM
DNA3	1.06 ± 0.06	0.089 ±0.009	0.9529	5	15 nM–0.3 mM	50 nM

## Data Availability

The data presented in this study are available in supplementary materials.

## Supplementary Materials

The following supporting information can be downloaded at: https://www.mdpi.com/article/10.3390/nano13162369/s1, Figure S1: The dependence of the polyPhTz peak currents on the incubation of the GCE/polyPhTz sensors in water and HEPES; cyclic voltammograms recorded with intermediate stirring the solution; Figure S2: The dependence of the polyPhTz peak currents on the incubation time; Figure S3: Cyclic voltammograms recorded after incubation of the GCE/polyPhTz/DNA sensor in doxorubicin solution; Figure S4: Anodic peak currents on cyclic voltammograms recorded after incubation of the GCE/polyPhTz/DNA sensor in doxorubicin solution within the storage period in the buffer and in dry conditions; Table S1: Analytical characteristics of the determination of doxorubicin with electrochemical sensors and DNA sensors. References [72,73,74,75,76,77,78,79,80,81,82,83,84,85,86,87] are cited in the supplementary materials.

## References

[1] (2019). World Health Organization Model List of Essential Medicines: 21st List 2019.

[2] Minotti G., Menna P., Salvatorelli E., Cairo G., Gianni L. (2004). Anthracyclines: Molecular advances and pharmacologic developments in antitumor activity and cardiotoxicity. Pharmacol. Rev..

[3] Berthiaume J.M., Wallace K.B. (2007). Adriamycin-induced oxidative mitochondrial cardiotoxicity. Cell Biol. Toxicol..

[4] Chatterjee K., Zhang J., Honbo N., Karliner J.S. (2010). Doxorubicin cardiomyopathy. Cardiology.

[5] Swain S.M., Whaley F.S., Ewer M.S. (2003). Congestive heart failure in patients treated with doxorubicin: A retrospective analysis of three trials. Cancer.

[6] Kondori T., Tajik S., Akbarzadeh T.N., Beitollahi H., Graiff C. (2022). Screen-printed electrode modified with co-NPs, as an electrochemical sensor for simultaneous determination of doxorubicin and dasatinib. J. Iran. Chem. Soc..

[7] Zhang W., Ma R., Gu S., Zhang L., Li N., Qiao J. (2022). Nitrogen and phosphorus co-doped carbon dots as an effective fluorescence probe for the detection of doxorubicin and cell imaging. Opt. Mater..

[8] Del Bonis-O’Donnell J.T., Pinals R.L., Jeong S., Thakrar A., Wolfinger R.D., Landry M.P. (2019). Chemometric approaches for developing infrared nanosensors to image anthracyclines. Biochemistry.

[9] Fleury-Souverain S., Maurin J., Guillarme D., Rudaz S., Bonnabry P. (2022). Development and application of a liquid chromatography coupled to mass spectrometry method for the simultaneous determination of 23 antineoplastic drugs at trace levels. J. Pharm. Biomed. Anal..

[10] Haq N., Alanazi F.K., Salem-Bekhit M.M., Rabea S., Alam P., Alsarra I.A., Shakeel F. (2022). Greenness estimation of chromatographic assay for the determination of anthracycline-based antitumor drug in bacterial ghost matrix of salmonella typhimurium. Sustain. Chem. Pharm..

[11] Estakhri N.M., Edwards B., Engheta N. (2019). Inverse-designed metastructures that solve equations. Science.

[12] Lalegani Z., Ebrahimi S.A.S., Hamawandi B., La Spada L., Batili H., Toprak M.S. (2022). Targeted dielectric coating of silver nanoparticles with silica to manipulate optical properties for metasurface applications. Mater. Chem. Phys..

[13] Lalegani Z., Ebrahimi S.A.S., Hamawandi B., La Spada L., Toprak M.S. (2020). Modeling, design, and synthesis of gram-scale monodispersed silver nanoparticles using microwave-assisted polyol process for metamaterial applications. Opt. Mater..

[14] Pacheco-Peña V., Beruete M., Rodríguez-Ulibarri P., Engheta N. (2019). On the performance of an ENZ-based sensor using transmission line theory and effective medium approach. New J. Phys..

[15] Akbari M., Shahbazzadeh M.J., La Spada L., Khajehzadeh A. (2021). The graphene field effect transistor modeling based on an optimized ambipolar virtual source model for DNA detection. Appl. Sci..

[16] Zhao H., Shi K., Zhang C., Ren J., Cui M., Li N., Ji X., Wang R. (2022). Spherical COFs decorated with gold nanoparticles and multiwalled carbon nanotubes as signal amplifier for sensitive electrochemical detection of doxorubicin. Microchem. J..

[17] Mehmandoust M., Khoshnavaz Y., Karimi F., Çakar S., Özacar M., Erk N. (2022). A novel 2-dimensional nanocomposite as a mediator for the determination of doxorubicin in biological samples. Environ. Res..

[18] Cunha C.E.P., Rodrigues E.S.B., De Oliveira Neto J.R., Somerset V., Taveira S., Sgobbi L., De Souza Gil E. (2022). Voltammetric glassy carbon sensor approach for the extended stability studies of doxorubicin in lyophilized dosage form. Eclet. Quim..

[19] Berneth H., Bayer A.G. (2003). Ullmann’s Encyclopedia of Industrial Chemistry.

[20] Brown J.S. (2022). Treatment of cancer with antipsychotic medications: Pushing the boundaries of schizophrenia and cancer. Neurosci. Biobehav. Rev..

[21] Mitchell S.C. (2006). Phenothiazine: The parent molecule. Curr. Drug Targets.

[22] Stanković D., Dimitrijević T., Kuzmanović D., Krstić M.P., Petković B.B. (2015). Voltammetric determination of an antipsychotic agent trifluoperazine at a boron-doped diamond electrode in human urine. RSC Adv..

[23] Vinothkumar V., Sakthivel R., Chen S.-M. (2022). Rare earth dysprosium nickelate nanospheres for the selective electrochemical detection of antipsychotic drug perphenazine in biological samples. Mater. Today Chem..

[24] Pulikkutty S., Manjula N., Chen T.-W., Chen S.-M., Lou B.-S., Siddiqui M.R., Wabaidur S.M., Ali M.A. (2022). Fabrication of gadolinium zinc oxide anchored with functionalized-SWCNT planted on glassy carbon electrode: Potential detection of psychotropic drug (phenothiazine) in biotic sample. J. Electroanal. Chem..

[25] Abad-Gil L., Brett C.M.A. (2022). Poly(methylene blue)-ternary deep eutectic solvent/Au nanoparticle modified electrodes as novel electrochemical sensors: Optimization, characterization and application. Electrochim. Acta.

[26] Manusha P., Yadav S., Satija J., Senthilkumar S. (2021). Designing electrochemical NADH sensor using silver nanoparticles/phenothiazine nanohybrid and investigation on the shape dependent sensing behavior. Sens. Actuators B.

[27] Sree V.G., Sohn J.I., Im H. (2022). Pre-anodized graphite pencil electrode coated with a poly(thionine) film for simultaneous sensing of 3-nitrophenol and 4-nitrophenol in environmental water samples. Sensors.

[28] Li Y., Liu D., Meng S., Dong N., Liu C., Wei Y., You T. (2022). Signal-enhanced strategy for ratiometric aptasensing of aflatoxin B1: Plasmon-modulated competition between photoelectrochemistry-driven and electrochemistry-driven redox of methylene blue. Biosens. Bioelectron..

[29] Goida A., Kuzin Y., Evtugyn V., Porfireva A., Evtugyn G., Hianik T. (2022). Electrochemical sensing of idarubicin—DNA interaction using electropolymerized azure B and methylene blue mediation. Chemosensors.

[30] Ivanov A., Stoikov D., Shafigullina I., Shurpik D., Stoikov I., Evtugyn G. (2022). Flow-through acetylcholinesterase sensor with replaceable enzyme reactor. Biosensors.

[31] Barsan M.M., Ghica M.E., Brett C.M.A. (2015). Electrochemical sensors and biosensors based on redox polymer/carbon nanotube modified electrodes: A review. Anal. Chim. Acta.

[32] Porfireva A., Plastinina K., Evtugyn V., Kuzin Y., Evtugyn G. (2021). Electrochemical DNA sensor based on poly(Azure A) obtained from the buffer saturated with chloroform. Sensors.

[33] Stoikov D.I., Porfir’eva A.V., Shurpik D.N., Stoikov I.I., Evtyugin G.A. (2019). Electrochemical DNA sensors on the basis of electropolymerized thionine and Azure B with addition of pillar[5]arene as an electron transfer mediator. Russ. Chem. Bull..

[34] Diaz A.F., Logan J.A. (1980). Electroactive polyaniline films. J. Electroanal. Chem..

[35] Diaz A.F., Kanazawa K.K., Gardini G.P. (1979). Electrochemical polymerization of pyrrole. J. Chem. Soc. Chem. Commun..

[36] Barsan M.M., Pinto E.M., Brett C.M.A. (2008). Electrosynthesis and electrochemical characterisation of phenazine polymers for application in biosensors. Electrochim. Acta.

[37] Topçu E., Alanyalıoğlu M. (2014). Electrochemical formation of poly(thionine) thin films: The effect of amine group on the polymeric film formation of phenothiazine dyes. J. Appl. Polym. Sci..

[38] Gligor D., Dilgin Y., Popescu I.C., Gorton L. (2009). Poly-phenothiazine derivative-modified glassy carbon electrode for NADH electrocatalytic oxidation. Electrochim. Acta.

[39] Puskás Z., Inzelt G. (2005). Formation and redox transformations of polyphenazine. Electrochim. Acta.

[40] Karyakin A.A., Cosnier S., Karyakin A. (2010). Chapter 5. Electropolymerized azines: A new group of electroactive polymers. Electropolymerization: Concepts, Materials and Applications.

[41] Seifi A., Afkhami A., Madrakian T. (2022). Highly sensitive and simultaneous electrochemical determination of lead and cadmium ions by poly(thionine)/MWCNTs-modified glassy carbon electrode in the presence of bismuth ions. J. Appl. Electrochem..

[42] Prado N.S., Silva L.A.J., Takeuchi R.M., Richter E.M., Santos A.L.D., Falcão E.H.L. (2022). Graphite sheets modified with poly(methylene blue) films: A cost-effective approach for the electrochemical sensing of the antibiotic nitrofurantoin. Microchem. J..

[43] Soranzo T., Ben Tahar A., Chmayssem A., Zelsmann M., Vadgama P., Lenormand J.-L., Cinquin P., Martin D.K., Zebda A. (2022). Electrochemical biosensing of glucose based on the enzymatic reduction of glucose. Sensors.

[44] Jiménez-Fiérrez F., González-Sánchez M.I., Jiménez-Pérez R., Iniesta J., Valero E. (2020). Glucose biosensor based on disposable activated carbon electrodes modified with platinum nanoparticles electrodeposited on poly(Azure A). Sensors.

[45] Fang J., Qi B., Yang L., Guo L. (2010). Ordered mesoporous carbon functionalized with poly-azure B for electrocatalytic application. J. Electroanal. Chem..

[46] Lin K.-C., Yin C.-Y., Chen S.-M. (2011). An electrochemical biosensor for determination of hydrogen peroxide using nanocomposite of poly(methylene blue) and FAD hybrid film. Sens. Actuators B.

[47] Ding M., Hou T., Niu H., Zhang N., Guan P., Hu X. (2022). Electrocatalytic oxidation of NADH at graphene-modified electrodes based on electropolymerized poly(thionine-methylene blue) films from nature deep eutectic solvents. J. Electroanal. Chem..

[48] Tiravia M., Sabuzi F., Cirulli M., Pezzola S., Di Carmine G., Cicero D.O., Floris B., Conte V., Galloni P. (2019). 3,7-Bis(N-methyl-N-phenylamino)phenothiazinium salt: Improved synthesis and aggregation behavior in solution. Eur. J. Org. Chem..

[49] Peterson B.M., Shen L., Lopez G.J., Gannett C.N., Ren D., Abruña H.D., Fors B.P. (2019). Elucidation of the electrochemical behavior of phenothiazine-based polyaromatic amines. Tetrahedron.

[50] Padnya P.L., Khadieva A.I., Stoikov I.I. (2022). Current achievements and perspectives in synthesis and applications of 3,7-disubstituted phenothiazines as methylene blue analogues. Dyes Pigment..

[51] Khadieva A., Rayanov M., Shibaeva K., Piskunov A., Padnya P., Stoikov I. (2022). Towards asymmetrical methylene blue analogues: Synthesis and reactivity of 3-N′-arylaminophenothiazines. Molecules.

[52] Wainwright M., McLean A. (2017). Rational design of phenothiazinium derivatives and photoantimicrobial drug discovery. Dyes Pigment..

[53] Kuzin Y.I., Padnya P.L., Stoikov I.I., Gorbatchuk V.V., Stoikov D.I., Khadieva A.I., Evtugyn G.A. (2020). Electrochemical behavior of the monomeric and polymeric forms of N-phenyl-3-(phenylimino)-3H-phenothiazin-7-amine. Electrochim. Acta.

[54] Salehan P., Ensafi A.A., Mousaabadi K.Z., Ghasemi J.B., Aghaee E., Rezaei B. (2022). A theoretical and experimental study of polyaniline/GCE and DNA G-quadruplex conformation as an impedimetric biosensor for the determination of potassium ions. Chemosphere.

[55] Ramanavicius S., Deshmukh M.A., Apetrei R.-M., Ramanaviciene A., Plikusiene I., Morkvenaite-Vilkonciene I., Thorat H.N., Shirsat M.D., Ramanavicius A. (2022). Chapter 15—Conducting polymers—Versatile tools in analytical systems for the determination of biomarkers and biologically active compounds. The detection of biomarkers: Past, present, and the future prospects. The Detection of Biomarkers. Past, Present and the Future Prospects.

[56] Tran L.T., Tran H.V., Cao H.H., Tran T.H., Huynh C.D. (2022). Electrochemically effective surface area of a polyaniline nanowire-based platinum microelectrode and development of an electrochemical DNA sensor. J. Nanotechnol..

[57] Khadieva A., Gorbachuk V., Shurpik D., Stoikov I. (2019). Synthesis of tris-pillar[5]arene and its association with phenothiazine dye: Colorimetric recognition of anions. Molecules.

[58] Evtugyn G., Porfireva A., Hianik T., Tiwari A., Patra H.K., Turner A.P.F. (2015). Electropolymerized materials for biosensors. Advanced Bioelectronics Materials.

[59] Kuzin Y.I., Khadieva A.I., Padnya P.L., Khannanov A.A., Kutyreva M.P., Stoikov I.I., Evtugyn G.A. (2021). Electrochemistry of new derivatives of phenothiazine: Electrode kinetics and electropolymerization conditions. Electrochim. Acta.

[60] Suprun E.V., Kutdusova G.R., Khmeleva S.A., Radko S.P. (2021). Towards deeper understanding of DNA electrochemical oxidation on carbon electrodes. Electrochem. Commun..

[61] Brett C.M.A. (2022). Electrochemical impedance spectroscopy in the characterisation and application of modified electrodes for electrochemical sensors and biosensors. Molecules.

[62] Kuzin Y., Kappo D., Porfireva A., Shurpik D., Stoikov I., Evtugyn G., Hianik T. (2018). Electrochemical DNA sensor based on carbon black—Poly(neutral red) composite for detection of oxidative DNA damage. Sensors.

[63] Chatterjee N., Walker G.C. (2017). Mechanisms of DNA damage, repair, and mutagenesis. Environ. Mol. Mutagen..

[64] Malanina A.N., Kuzin Y.I., Ivanov A.N., Ziyatdinova G.K., Shurpik D.N., Stoikov I.I., Evtugyn G.A. (2022). Polyelectrolyte polyethylenimine-DNA complexes in the composition of voltammetric sensors for detecting DNA damage. J. Anal. Chem..

[65] Gunvén G., Theve N.O., Peterson C. (1986). Serum and tissue concentrations of doxorubicin after IV administration of doxorubicin or doxorubicin-DNA complex to patients with gastrointestinal cancer. Cancer Chemother. Pharmacol..

[66] Chai M., Wientjes M.G., Badalament R.A., Burgers J.K., Au J.L.-S. (1994). Pharmacokinetics of intra vesical doxorubicin in superficial bladder cancer patients. J. Urol..

[67] Krarup-Hansen A., Wassermann K., Rasmussen S.N., Dalmark M. (1988). Pharmacokinetics of doxorubicin in man with induced acid or alkaline urine. Acta Oncol..

[68] Maudens K.E., Stove C.P., Lambert W.E. (2011). Quantitative liquid chromatographic analysis of anthracyclines in biological fluids. J. Chromatogr. B.

[69] Shamagsumova R., Porfireva A., Stepanova V., Osin Y., Evtugyn G., Hianik T. (2015). Polyaniline–DNA based sensor for the detection of anthracycline drugs. Sens. Actuators B.

[70] Hajian R., Mehrayin Z., Mohagheghian M., Zafari M., Hosseini P., Shams N. (2015). Fabrication of an electrochemical sensor based on carbon nanotubes modified with gold nanoparticles for determination of valrubicin as a chemotherapy drug: Valrubicin-DNA interaction. Mater. Sci. Eng. C.

[71] Hongpaisan J., Roomans G.M. (1999). Retaining ionic concentrations during in vitro storage of tissue for microanalytical studies. J. Microsc..

[72] Alavi-Tabari S.A.R., Khalilzadeh M.A., Karimi-Maleh H. (2018). Simultaneous determination of doxorubicin and dasatinib as two breast anticancer drugs uses an amplified sensor with ionic liquid and ZnO nanoparticle. J. Electroanal. Chem..

[73] Liu J., Bo X., Zhou M., Guo L. (2019). A nanocomposite prepared from metal-free mesoporous carbon nanospheres and graphene oxide for voltammetric determination of doxorubicin. Microchim. Acta.

[74] Vacek J., Havran L., Fojta M. (2009). Ex situ voltammetry and chronopotentiometry of doxorubicin at a pyrolytic graphite elec-trode: Redox and catalytic properties and analytical applications. Electroanalysis.

[75] Skalová Š., Langmaier J., Barek J., Vyskočil V., Navrátils T. (2020). Doxorubicin determination using two novel voltammetric approaches: A comparative study. Electrochim. Acta.

[76] Ali A.-M.B.H., Rageh A.H., Abdel-aal F.A.M., Mohamed A.-M.I. (2023). Anatase titanium oxide nanoparticles and multi-walled carbon nanotubes-modified carbon paste electrode for simultaneous determination of avanafil and doxorubicin in plasma samples. Microchem. J..

[77] Singh T.A., Sharma V., Thakur N., Tejwan N., Sharma A., Das J. (2023). Selective and sensitive electrochemical detection of doxorubicin via a novel magnesium oxide/carbon dot nanocomposite based sensor. Inorg. Chem. Commun..

[78] Abbasi M., Ezazi M., Jouyban A., Lulek E., Asadpour-Zeynali K., Ertas Y.N., Houshyar J., Mokhtarzadeh A., Soleymani J. (2022). An ultrasensitive and preprocessing-free electrochemical platform for the detection of doxorubicin based on tryptophan/polyethylene glycol-cobalt ferrite nanoparticles modified electrodes. Microchem. J..

[79] Porfireva A., Vorobev V., Babkina S., Evtugyn G. (2019). Electrochemical sensor based on poly(Azure B)-DNA composite for doxorubicin determination. Sensors.

[80] Kulikova T., Porfireva A., Evtugyn G., Hianik T. (2019). Electrochemical DNA sensors with layered polyaniline-DNA coating for detection of specific DNA interactions. Sensors.

[81] Peng A., Xu H., Luo C., Ding H. (2016). Application of a disposable doxorubicin sensor for direct determination of clinical drug concentration in patient blood. Int. J. Electrochem. Sci..

[82] Evtugyn G., Porfireva A., Stepanova V., Budnikov H. (2015). Electrochemical biosensors based on native DNA and nanosized mediator for the detection of anthracycline preparations. Electroanalysis.

[83] Kulikova T., Porfireva A., Rogov A., Evtugyn G. (2021). Electrochemical DNA sensor based on acridine yellow adsorbed on glassy carbon electrode. Sensors.

[84] Kappo D., Shurpik D., Padnya P., Stoikov I., Rogov A., Evtugyn G. (2022). Electrochemical DNA sensor based on carbon black-poly(methylene blue)-poly(neutral red) composite. Biosensors.

[85] Karadurmus L., Dogan-Topal B., Kurbanoglu S., Shah A., Ozkan S.A. (2021). The interaction between DNA and three intercalating anthracyclines using electrochemical DNA nanobiosensor based on metal nanoparticles modified screen-printed electrode. Micromachines.

[86] Moghadam F.H., Taher M.A., Karimi-Maleh H. (2021). Doxorubicin anticancer drug monitoring by ds-DNA-based electrochemical biosensor in clinical samples. Micromachines.

[87] Asai K., Yamamoto T., Nagashima S., Ogata G., Hibino H., Einaga Y. (2020). An electrochemical aptamer-based sensor prepared by utilizing the strong interaction between a DNA aptamer and diamond. Analyst.

