# Supplementary material for: Voltammetric Sensor for Doxorubicin Determination Based on Self-Assembled DNA-Polyphenothiazine Composite
Source: Nanomaterials (Basel). 2023 Aug 18;13(16):2369. doi: 10.3390/nano13162369 (PMC10459114; doi:10.3390/nano13162369)
Supplement: Supplementary file 1 [file nanomaterials-13-02369-s001.zip › nanomaterials-2544356-supplementary.pdf]

nanomaterials

Supplementary Materials

Voltammetric Sensor for Doxorubicin Determination Based on Self-Assembled DNA-Polyphenothiazine Composite

Anastasiya Malanina [1], Yurii Kuzin [1], Alena Khadieva [1], Kseniya Shibaeva [1], Pavel Padnya [1,*], Ivan Stoikov [1] and Gennady Evtugyn [1,2,*]

[1] A.M. Butlerov' Chemistry Institute, Kazan Federal University, 18 Kremlevskaya Street, Kazan 420008, Russia; malanast@mail.ru (A.M.); yzinkyra@mail.ru (Y.K.); aljoihadieva@kpfu.ru (A.K.); ksshibaeva@kpfu.ru (K.S.); ivan.stoikov@mail.ru (I.S.)

[2] Analytical Chemistry Department, Chemical Technology Institute, Ural Federal University, 19 Mira Street, Ekaterinburg 620002, Russia

* Correspondence: padnya.ksu@gmail.com (P.P.); gennady.evtugyn@kpfu.ru (G.E.)

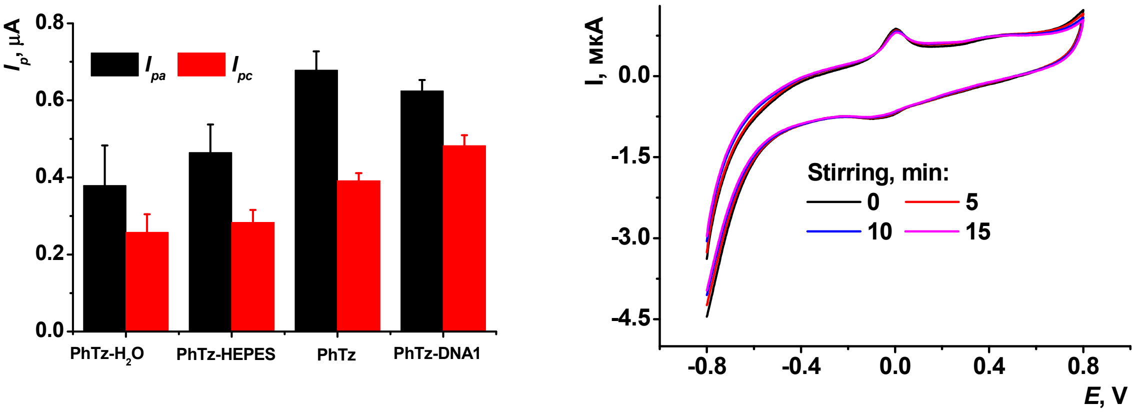

(a) (b)

Figure S1. (**a**) The dependence of the polyPhTz peak currents on the incubation of the GCE/polyPhTz sensors in water and HEPES. Average ± S.D. for five sensors, incubation 20 min; (**b**) Cyclic voltammograms recorded in 0.1 M HEPES + 0.1 M $NaNO_3$, pH 7.0, scan rate 100 mV/s, with intermediate stirring the solution.

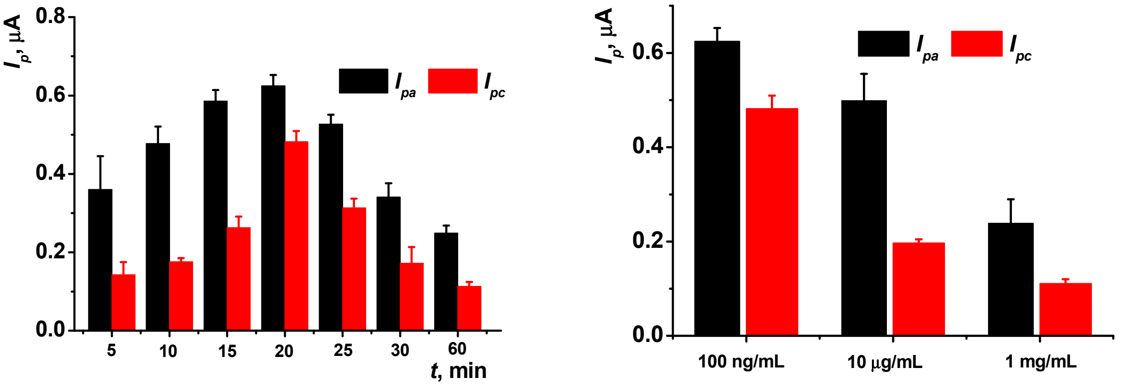

(a) (b)

Figure S2. (**a**) The dependence of the polyPhTz peak currents on the incubation time of the GCE/polyPhTz sensors in 0.1 M HEPES; (**b**) The polyPhTz peak currents after 15 min incubation of the GCE/polyPhTz sensor in DNA1 solution of various concentration. Average ± S.D. for five sensors.

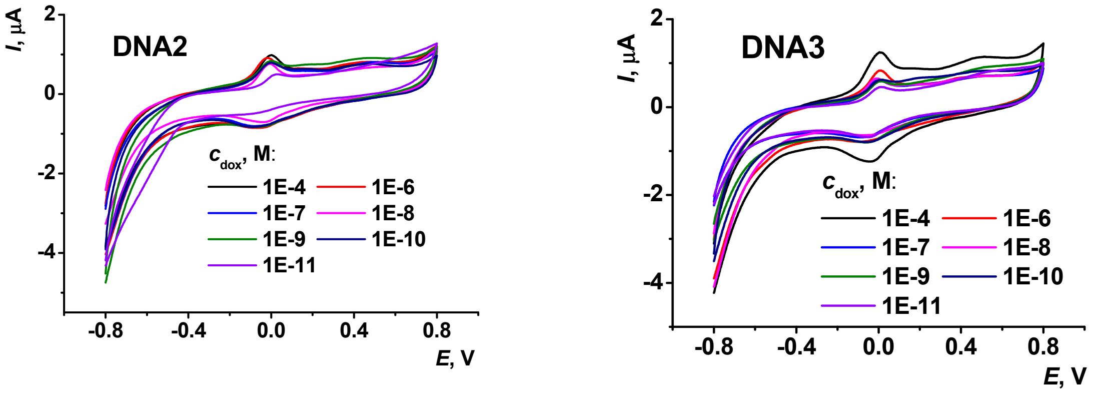

(a) (b)

Figure S3. Cyclic voltammograms recorded after incubation of the GCE/polyPhTz/DNA sensor in doxorubicin solution. (**a**) DNA from fish sperm; (**b**) DNA from chicken erythrocytes.

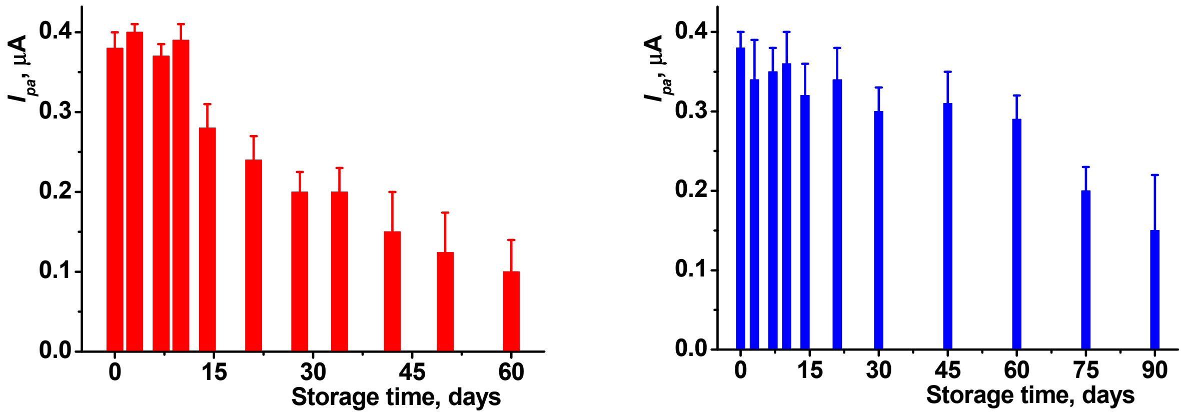

(a) (b)

Figure S4. Anodic peak currents on cyclic voltammograms recorded after incubation of the GCE/polyPhTz/DNA (0.1 mg/mL DNA) sensor in 0.1 nM doxorubicin solution. (**a**) Storage in 0.1 M HEPES buffer at 4 °C; (**b**)Storage in dry conditions at 4 °C. Average ± S.D. for five individual sensors

Table 1. Analytical characteristics of the determination of doxorubicin with electrochemical sensors and DNA sensors.

Electrode / Modifier	Concentration range, µM	LOD, nM	Ref
Electrochemical sensors			
ZnO /graphite paste electrode	0.07–5000	9.0	[s1]
GCE/mesoporous carbon nanospheres / reduced graphene oxide	0.01–10	1.5	[s2]
Pyrographite	0.01–1.0	10	[s3]
Pt/ Silver solid amalgam	0.6–10	440	[s4]
Carbon paste electrode with implemented TiO_2 nanoparticles and multi-walled carbon nanotubes	5.0–35.0	1300	[s5]
Screen-printed carbon electrode /MgO/carbon nanodots/	0.1–1.0	90	[s6]
GCE/ Tryptophan / polyethylene glycol / $CoFe_2O_4$ nanoparticles	0.06–2.0	30	[s7]
Electrochemical DNA sensors			

GCE / poly(Azure B)	0.0001–0.1	0.07	[s8]
GCE/Polyaniline/DNA	$1\cdot10^{-6}$–1000	0.0006	[s9]
GCE / carbon nanotubes–polylysine	0.0025–0.25	1.0	[s10]
GCE / poly(Neutral red)	0.0001–0.1	50	[s11]
GCE / acridine yellow (monomer)	$1\cdot10^{-5}$–0.001	0.7	[s12]
GCE—poly(Methylene blue) -poly(Neutral red)	0.0005–1000	0.13	[s13]
Screen-printed carbon electrode/ Pt nanoparticles / Ag nanoparticles / DNA	0.2–2.0	-	[s14]
GCE / Single-walled carbon nanotubes	0.001–20	0.6	[s15]
Boron doped diamond electrode / DNA aptamer	Up to 2.3	49	[s16]
GCE/PolyPhTz/DNA	0.01–200	0.005	This work

[s1] Alavi-Tabari, S.A.R.; Khalilzadeh, M.A.; Karimi-Maleh, H. Simultaneous determination of doxorubicin and dasatinib as two breast anticancer drugs uses an amplified sensor with ionic liquid and ZnO nanoparticle. *J. Electroanal. Chem.* **2018**, *811*, 84–88. doi:10.1016/j.jelechem.2018.01.034.

[s2] Liu, J.; Bo, X.; Zhou, M.; Guo, L. A nanocomposite prepared from metal-free mesoporous carbon nanospheres and graphene oxide for voltammetric determination of doxorubicin. *Microchim. Acta* **2019**, *186*, 639, doi:10.1007/s00604-019-3754-5

[s3] Vacek, J.; Havran, L.; Fojta, M. Ex situ voltammetry and chronopotentiometry of doxorubicin at a pyrolytic graphite elec-trode: Redox and catalytic properties and analytical applications. *Electroanalysis* **2009**, *21*, 21399–22144, doi:10.1002/elan.200904646.

[s4] Skalová, Š.; Langmaier, J.; Barek, J.; Vyskočil, V.; Navrátils, T. Doxorubicin determination using two novel voltammetric approaches: A comparative study. *Electrochim. Acta* **2020**, *330*, 135180. doi: 10.1016/j.electacta.2019.135180.

[s5] Ali, A.-M.B.H.; Rageh, A.H.; Abdel-aal, F.A.M.; Mohamed, A.-M.I. Anatase titanium oxide nanoparticles and multi-walled carbon nanotubes-modified carbon paste electrode for simultaneous determination of avanafil and doxorubicin in plasma samples. *Microchem. J.* **2023**, *185*, 108261. https://doi.org/10.1016/j.microc.2022.108261.

[s6] Singh, T.A.; Sharma, V.; Thakur, N.; Tejwan, N.; Sharma, A.; Das, J. Selective and sensitive electrochemical detection of doxorubicin via a novel magnesium oxide/carbon dot nanocomposite based sensor. *Inorg. Chem. Commun.* **2023**, *150*, 110527. https://doi.org/10.1016/j.inoche.2023.110527.

[s7] Abbasi, M., Ezazi, M., Jouyban, A., Lulek, E.; Asadpour-Zeynali, K.; Ertas, Y.N.; Houshyar, J.; Mokhtarzadeh, A., Soleymani, J. An ultrasensitive and preprocessing-free electrochemical platform for the detection of doxorubicin based on tryptophan/polyethylene glycol-cobalt ferrite nanoparticles modified electrodes. Microchem. J. 2022, 183, 108055. https://doi.org/10.1016/j.microc.2022.108055.

[s8] Porfireva, A.; Vorobev, V.; Babkina, S.; Evtugyn, G. Electrochemical sensor based on poly(Azure B)-DNA composite for doxorubicin determination. *Sensors* **2019**, *19*, 2085. doi: 10.3390/s19092085.

[s9] Kulikova, T.; Porfireva, A.; Evtugyn, G.; Hianik, T. Electrochemical DNA sensors with layered polyaniline-DNA coating for detection of specific DNA interactions. *Sensors* **2019**, *19*, 469, doi:10.3390/s19030469.

[s10] Peng, A.; Xu, H.; Luo, C.; Ding, H. Application of a disposable doxorubicin sensor for direct determination of clinical drug concentration in patient blood. *Int. J. Electrochem. Sci.* **2016**, 11, 6266–6278, doi:10.20964/2016.07.38.

[s11] Evtugyn, G.; Porfireva, A.; Stepanova, V.; Budnikov, H. Electrochemical biosensors based on native DNA and nanosized mediator for the detection of anthracycline preparations. *Electroanalysis* **2015**, *27*, 629-637. DOI: 10.1002/elan.201400564.

[s12] Kulikova,T.; Porfireva, A.; Rogov, A.; Evtugyn, G. Electrochemical DNA sensor based on acridine yellow adsorbed on glassy carbon electrode. *Sensors* **2021**, *21*, 7763. DOI: 10.3390/s21227763.
[s13] Kappo, D.; Shurpik, D.; Padnya, P.; Stoikov, I.; Rogov, A.; Evtugyn, G. Electrochemical DNA sensor based on carbon black-poly(methylene blue)-poly(neutral red) composite. *Biosensors* **2022**, *12*, 329. DOI: 10.3390/bios12050329
[s14] Karadurmus, L.; Dogan-Topal, B.; Kurbanoglu, S.; Shah, A.; Ozkan, S.A. The interaction between DNA and three intercalating anthracyclines using electrochemical DNA nanobiosensor based on metal nanoparticles modified screen-printed electrode. *Micromachines* **2021**, *12*, 1337; https://doi.org/10.3390/mi12111337.
[s15] Moghadam, F. H.; Taher, M.A.; Karimi-Maleh, H. Doxorubicin anticancer drug monitoring by ds-DNA-based electrochemical biosensor in clinical samples. *Micromachines* **2021**, *12*, 808; doi: 10.3390/mi12070808.
[s16] Asai, K.; Yamamoto, T.; Nagashima, S.; Ogata, G.; Hibino, H.; Einaga, Y. An electrochemical aptamer-based sensor prepared by utilizing the strong interaction between a DNA aptamer and diamond. *Analyst* **2020**, *145*, 544–549. DOI: 10.1039/c9an01976
